# Red Cell Distribution Width Is Associated with All-Cause and Cardiovascular Mortality in Patients with Diabetes

**DOI:** 10.1155/2017/5843702

**Published:** 2017-11-21

**Authors:** Sadeer G. Al-Kindi, Marwan Refaat, Amin Jayyousi, Nidal Asaad, Jassim Al Suwaidi, Charbel Abi Khalil

**Affiliations:** ^1^Harrington Heart and Vascular Institute, University Hospitals Cleveland Medical Center, Cleveland, OH, USA; ^2^Department of Internal Medicine, Cardiovascular Medicine/Cardiac Electrophysiology, American University of Beirut Faculty of Medicine and Medical Center, Beirut, Lebanon; ^3^Department of Diabetes, Hamad Medical Corporation, Doha, Qatar; ^4^Adult Cardiology, Heart Hospital, Hamad Medical Corporation, Doha, Qatar; ^5^Department of Medicine and Genetic Medicine, Weill Cornell Medicine, Doha, Qatar

## Abstract

**Background and Methods:**

Red cell distribution width (RDW) has emerged as a prognostic marker in patients with cardiovascular diseases. We investigated mortality in patients with diabetes included in the National Health and Nutrition Examination Survey, in relation to baseline RDW. RDW was divided into 4 quartiles (Q1: ≤12.4%, Q2: 12.5%–12.9%, Q3: 13.0%–13.7%, and Q4: >13.7%).

**Results:**

A total of 3,061 patients were included: mean age 61 ± 14 years, 50% male, 39% White. Mean RDW was 13.2% ± 1.4%. Compared with first quartile (Q1) of RDW, patients in Q4 were more likely to be older, female, and African-American, have had history of stroke, myocardial infarction, and heart failure, and have chronic kidney disease. After a median follow-up of 6 years, 628 patient died (29% of cardiovascular disease). Compared with Q1, patients in Q4 were at increased risk for all-cause mortality (HR 3.44 [2.74–4.32], *P* < .001) and cardiovascular mortality (HR 3.34 [2.16–5.17], *P* < .001). After adjusting for 17 covariates, RDW in Q4 remained significantly associated with all-cause mortality (HR 2.39 [1.30–4.38], *P* = 0.005) and cardiovascular mortality (HR 1.99 [1.17–3.37], *P* = 0.011).

**Conclusion:**

RDW is a powerful and an independent marker for prediction of all-cause mortality and cardiovascular mortality in patients with diabetes.

## 1. Introduction

Diabetes is associated with increased risk of microvascular and macrovascular complications [1–3]. Cardiovascular disease (CVD) is the leading cause of death among patients with diabetes accounting for 30–40% of deaths [4–6]. The risk of CVD can be modified using pharmacologic and nonpharmacologic measures [7–11]. Thus, it is important to accurately estimate the risk of cardiovascular disease to allocate resources and focus preventive measures among these high risk patients. While many risk scores have been devised to estimate the risk of cardiovascular disease among patients with diabetes, they often are difficult to incorporate in the clinical routine and have only modest discriminatory power [12, 13].

Red cell distribution width (RDW), a measure of variability in red blood cell size, is routinely measured in complete blood counts and is traditionally used to identify etiology of anemia. It is automatically calculated as standard deviation of mean corpuscular volume divided by mean corpuscular volume × 100%. Over the past decade, RDW has emerged as a prognostic marker in patients with CVD. Several studies have reported the prognostic power of RDW in patients with heart failure (HF) [14, 15] and coronary artery disease (CAD) [16–18], where it appears to be a powerful and independent marker of outcomes. Additionally, RDW has also been shown to predict incident diabetes [19, 20], incident CVD, and mortality in community-dwelling subjects [21, 22].

Patients with diabetes have higher RDW than patients without diabetes [23, 24]. One prior study showed that, among patients with diabetes, RDW is associated with the presence of microvascular and macrovascular complications [25]. Whether RDW predicts mortality in patients with diabetes is not known. We sought to investigate the association between RDW with all-cause and cardiovascular mortality in a large representative cohort of noninstitutionalized patients with diabetes.

## 2. Methods

### 2.1. Dataset

NHANES is a program of studies designed to understand the health and nutritional status of adults and children in the US. This study was designed as a cross-sectional, repeated, multistage survey of noninstitutionalized US adults and children. This survey included questionnaires, physical examination, and laboratory testing. We included all adults (≥18 years) with self-reported diabetes mellitus, who were enrolled in the NHANES between 1999 and 2010, and have linkage to mortality data as described later (follow-up until 2011). All protocols were approved by the institutional review board at the National Center for Health Statistics (NCHS), and all participants provided informed consent.

### 2.2. Predictor Variable

Red cell distribution was measured from blood obtained from participants at the time of examination. RDW was measured using the Beckman Coulter MAXM instrument in the Mobile Examination Center. RDW was treated as continuous and categorical (quartile) variable in this analysis.

### 2.3. Outcomes

Mortality was identified through probabilistic linkage with the national death index using patient identifiers (e.g., social security number and date of birth) through 2011. The linkage is performed by the National Center for Health Statistics [26]. For this study, we identified all-cause mortality and cardiovascular mortality as defined by the 10th revision of International Classification of Diseases codes (I00 to I99).

### 2.4. Statistical Analyses

Continuous variables are presented as means (standard deviations) or median (25th–75th percentiles) as appropriate. Categorical variables are presented as numbers and percentages. No assumptions were made for missing variables. Logistic regression models were used to identify the association between RDW and the underlying comorbidities (self-reported myocardial infarction (MI), self-reported stroke, and chronic kidney disease (CKD): defined as estimated glomerular filtration rate (eGFR) of less than 60 ml/mins per 1.73 m^2^ using the CKD-EPI equation [27]), with adjustment for (defined a priori) age, gender, race, hemoglobin, SBP, smoking, cholesterol, and insulin use. Unadjusted survival analyses were performed with Kaplan-Meier method and compared using Log Rank (Mantel-Cox) test. The follow-up duration was estimated using the reverse Kaplan-Meier method described by Schemper and Smith [28]. Cox proportional hazard models were adjusted for the following covariables (defined a priori): model 1: age, gender, race, and hemoglobin; model 2: model 1 + HF, MI, stroke, malignancy, CKD, BMI, SBP, and cholesterol; model 3: model 2 + oral antidiabetics, insulin, statins, ACE/ARBs, and diuretics. Hazard ratios and 95% confidence intervals for the adjusted and unadjusted models were estimated using Cox proportional hazard models. Penalized smoothing splines were also performed using the spline and survival packages in R to visualize the association of continuous RDW with hazards of mortality. All tests were two-sided and *P* < 0.05 was considered statistically significant. All analyses were performed on Statistical Package for Social Sciences (SPSS, version 21) and R-Package 3.3.1 for Windows.

## 3. Results

**Figure 1:**
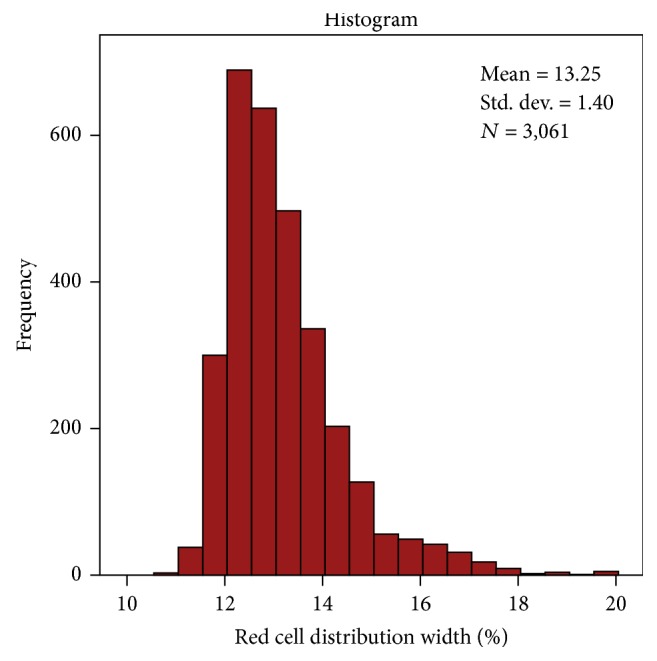
Distribution of red cell distribution width in the study cohort.

A total of 3061 patients were included: mean age 61 ± 14 years, 50% male, 39% White. Mean RDW was 13.2% ± 1.4%. Distribution of RDW is shown in Figure 1. Compared with first quartile (Q1) of RDW, patients in Q4 were more likely to be older (Q1 versus Q4, age 58 versus 65 years, *P* < 0.001), female (48% versus 57%, *P* < 0.001), and African Americans (13% versus 42%, *P* < 0.001), have had history of stroke (6% versus 15%, *P* < 0.001), MI (6% versus 20%, *P* < 0.001), and HF (3.8% versus 21%, *P* < 0.001), and have CKD (13% versus 35%, *P* < 0.001), albuminuria (median ACR 0.12 versus 0.25, *P* < 0.001), and higher c-reactive protein (0.24 versus 0.45 mg/dL, *P* < 0.001), but there was no difference in the prevalence of retinopathy (*P* = 0.77) (Table 1).

RDW correlated negatively with hemoglobin (*r* = −0.47, *P* < 0.001), mean red cell volume (*r* = −0.29, *P* < 0.001), and mean red cell hemoglobin (*r* = −0.37, *P* < 0.001), eGFR (*r* = −0.25, *P* < 0.001), and positively with c-reactive protein (*r* = 0.20, *P* < 0.001) and urine albumin : creatinine ratio (*r* = 0.15, *P* < 0.001).

RDW was associated with underlying diabetes-related complications (MI, stroke, and CKD). Compared with Q1 and after adjusting for age, gender, race, hemoglobin, SBP, smoking, cholesterol, and insulin use, patients in Q4 had higher risk of MI (OR 3.17 [2.17–4.64], *P* < 0.001), stroke (OR 1.12 [1.03–1.22], *P* = 0.006), and CKD (OR 1.13 [1.05–1.21], *P* = 0.002). Diabetes-related complications increased with RDW: MI (OR 1.22 [1.13–1.33] per 1% increment in RDW, *P* < 0.001), stroke (OR 1.12 [1.03–1.22] per 1% increment in RDW, *P* = 0.006), and CKD (OR 1.13 [1.05–1.21] per 1% increment in RDW, *P* = 0.002).  Table 2 shows the odds ratio of diabetes-related complications in unadjusted and adjusted models.

**Figure 2:**
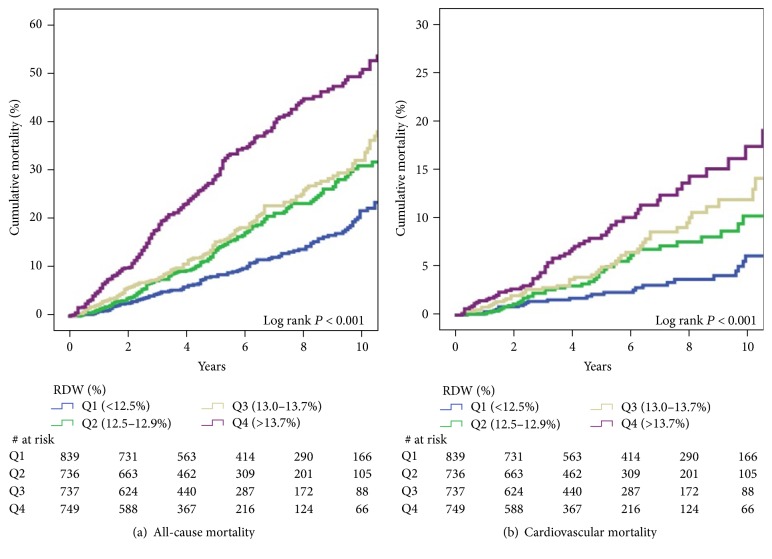
Kaplan-Meier curves of all-cause (a) and cardiovascular (b) mortality by quartiles of RDW. RDW: red cell width distribution.

**Figure 3:**
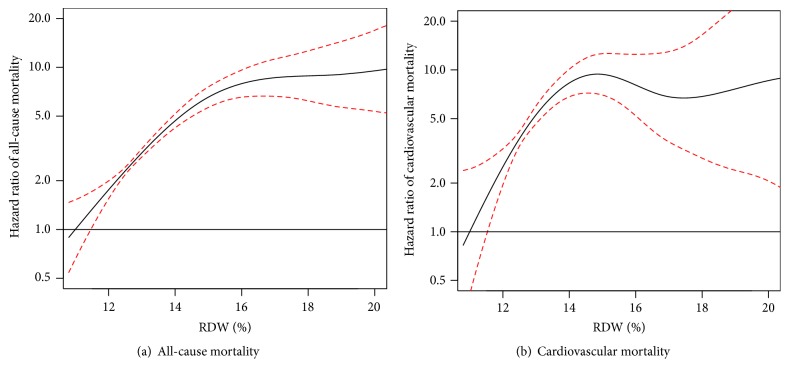
Association between continuous RDW with all-cause (a) and cardiovascular (b) mortality. RDW: red cell width distribution.

After a median follow-up of 6 years, 628 patients died (29% of CVD). Compared with Q1, patients in Q4 were at increased risk for all-cause mortality (HR 3.44 [2.74–4.32], *P* < 0.001) and cardiovascular mortality (HR 3.34 [2.16–5.17], *P* < 0.001).  Figure 2 depicts the Kaplan-Meier figures of all-cause and cardiovascular mortality by RDW quartile. After adjusting for 17 covariates, RDW in Q4 remained significantly associated with all-cause mortality (HR 2.39 [1.30–4.38], *P* = 0.005) and cardiovascular mortality (HR 1.99 [1.17–3.37], *P* = 0.011).  Table 3 shows the multivariable adjusted models by quartile of RDW for all-cause and cardiovascular mortality. In a penalized smoothing spline, and compared with RDW of 11%, hazard ratio of all-cause mortality and cardiovascular mortality increased significantly until about RDW of 15%, with no further increase with higher values, Figure 3.

## 4. Discussion

To our knowledge, this is the first study to evaluate the prognostic implications of RDW in community-dwelling patients with diabetes. We show that RDW is associated with underlying diabetes-related complications, namely, MI, stroke, and CKD. We also show that RDW is an independent and strong marker of cardiovascular and all-cause mortality in these patients.

Elevated RDW indicated high variability of erythrocyte size, which is a marker of ineffective erythropoiesis. Prior studies have identified an association between RDW and markers of inflammation such as Interleukin 6 [15], soluble tumor necrosis factor [29], iron mobilization (soluble transferrin receptor [15]), and oxidative stress [30]. All these mechanisms have been implicated in erythropoiesis and anemia. While higher RDW is associated with lower hemoglobin, in this analysis the mean hemoglobin across the 4 quartiles did not fall into the “anemia” range.

Our study confirms a prior analysis of the association between RDW and underlying diabetes-related complications in cross-sectional study design. In a study of 2,497 patients with diabetes enrolled in the previous version of NHANES (NHANES III, 1988–1994), third and fourth quartiles of RDW were associated with increased odds of myocardial infarction (OR 2.45 [95% CI 1.13, 5.28]), stroke (OR 2.56 [1.21–5.42]), and nephropathy (OR 2.33 [1.42–3.82]), but not retinopathy [25]. Another smaller study showed that RDW is independently associated with underlying microalbuminuria in patients newly diagnosed with diabetes [31]. Our study validates these observations in an independent cohort. Because of the cross-sectional design, however, the temporal relationship of these events cannot be ascertained.

The prognostic role of RDW in diabetes is incompletely understood. To our knowledge, only one study investigated the prognostic impact of RDW in patients with diabetes with CAD. Among 560 patients with diabetes and stable CAD who underwent percutaneous coronary intervention, high RDW (≥13.1%) was independently associated with all-cause mortality (HR 2.56 [1.12–6.62], *P* = 0.025) [32]. Our findings generalize the prognostic role of RDW in predicting not only all-cause mortality but also cardiovascular mortality in a larger cohort of patients with diabetes with low prevalence of cardiovascular disease. It is important to note that in our fully adjusted model (model 3, Table 3), only 4th quartile of RDW (>13.7%) was consistently associated with increased cardiovascular and all-cause mortality. This is likely related to a threshold effect within RDW that limits our conclusions in mid-range RDW (12.4%–13.7%), as these levels (quartiles 2 and 3) were only associated with cardiovascular and all-cause mortality in partially adjusted models.

We also show that the risk of cardiovascular and all-cause mortality increased with RDW at levels considered within the normal limit in many clinical laboratories. As shown in Figure 3, hazards of cardiovascular mortality and all-cause mortality start increasing at about RDW of 12%. It is thus important to reconsider the traditional cutoffs if this test is to be used for prognostic and cardiovascular risk predictions.

Measurement of RDW often incurs no additional cost as it is a part of the routine automated complete blood counts and can provide prognostic information beyond traditional factors. Future studies should investigate the incremental value of adding RDW to predictive risk scores for cardiovascular disease in patients with diabetes. RDW could be used to select a cohort of patients enriched for poor outcomes for prevention trials. As shown in Figure 3, RDW higher than 15% was associated with approximately 10-fold increase in mortality, thus serving as a powerful tool for risk stratification in this high risk group. The change in RDW could potentially serve as a surrogate marker for all-cause and cardiovascular mortality that could be used in pilot studies of primary and secondary prevention of cardiovascular disease in diabetes.

Our study has few limitations that need to be acknowledged. We lack vital data on the duration of diabetes, type of diabetes, and etiology, as well as the prevalence of other cardiovascular risk factors such as dyslipidemia or hypertension. Cause specific mortality is derived from death certificates and thus may not be accurate in classifying etiology, particularly in out-of-hospital deaths. The dataset also does not capture incident cardiovascular events, such as myocardial infarctions or strokes, that would be important to describe in relationship to RDW. Additionally, data on factors related to RDW such as nutritional deficiencies (e.g., iron, folate, or vitamin B12) or blood transfusions are not consistently available in the dataset.

## 5. Conclusion

Red cell distribution is a powerful and an independent prognostic marker for prediction of all-cause mortality and cardiovascular mortality in patients with diabetes. Further studies should focus on incorporating RDW in risk prediction models in diabetes.

## Figures and Tables

**Table 1:** Baseline characteristics of patients with diabetes by quartiles of RDW (NHANES 1999–2010).

Characteristics	Red cell distribution width (%)				*P* value^*∗*^
	Q1 (*n* = 839) ≤12.4%	Q2 (*n* = 736) 12.5%–12.9%	Q3 (*n* = 737) 13.0%–13.7%	Q4 (*n* = 749) >13.7%	
Age (years), mean ± SD	58 ± 14	62 ± 14	64 ± 12	65 ± 13	**<0.001**
Women, *n* (%)	401 (48%)	347 (47%)	351 (48%)	424 (57%)	**<0.001**
African-American, *n* (%)	109 (13%)	141 (19%)	213 (29%)	314 (42%)	**<0.001**
Ever smoker (%)	412 (50%)	372 (51%)	372 (51%)	408 (55%)	0.22
History of MI, *n* (%)	50 (6%)	73 (10%)	105 (14%)	147 (20%)	**<0.001**
History of HF, *n* (%)	31 (4%)	50 (7%)	91 (13%)	157 (21%)	**<0.001**
History of malignancy, *n* (%)	104 (13%)	85 (12%)	96 (13%)	130 (17%)	**0.017**
History of stroke, *n* (%)	50 (6%)	62 (9%)	89 (12%)	112 (15%)	**<0.001**
BMI (kg/m^2^), mean ± SD	30 ± 6	31 ± 7	33 ± 7	33 ± 9	<0.001
SBP (mmHg), mean ± SD	131 ± 21	134 ± 22	133 ± 21	135 ± 23	**0.001**
Hemoglobin (g/dL), mean ± SD	14.5 ± 1.4	14.1 ± 1.4	13.8 ± 1.4	12.8 ± 1.7	**<0.001**
eGFR (ml/min per 1.73 m^2^), mean ± SD	90 ± 24	83 ± 25	80 ± 26	71 ± 31	**<0.001**
CKD (eGFR < 60), *n* (%)	105 (13%)	143 (19%)	173 (24%)	262 (35%)	**<0.001**
Retinopathy, *n* (%)	183 (22%)	168 (23%)	165 (22%)	188 (25%)	0.77
UACR, median [IQR]	0.12 [0.06–0.37]	0.14 [0.07–0.44]	0.17 [0.07–0.61]	0.25 [0.08–0.96]	**<0.001**
Total cholesterol (mg/dL), mean ± SD	197 ± 45	192 ± 45	188 ± 44	188 ± 54	**<0.001**
Hemoglobin A1c (%), median [IQR]	7.1 [6.2–8.6]	7.0 [6.1–8.3]	6.9 [6.2–8.0]	6.8 [6.1–7.7]	<0.001
Random blood glucose (mg/dL), median [IQR]	139 [106–205]	136 [106–186]	128 [101–172]	125 [98–170]	<0.001
CRP (mg/dL), median [IQR]	0.24 [0.10–0.54]	0.25 [0.12–0.55]	0.34 [0.14–0.74]	0.45 [0.20–1.03]	**<0.001**
Medications					
Oral antidiabetic, *n* (%)	532 (63%)	515 (70%)	530 (72%)	520 (69%)	0.002
Metformin, *n* (%)	334 (40%)	354 (48%)	357 (48%)	309 (41%)	<0.001
Insulin, *n* (%)	185 (22%)	167 (23%)	208 (28%)	234 (31%)	**<0.001**
Aspirin, *n* (%)	35 (4%)	29 (4%)	39 (5%)	40 (5%)	0.43
ACE/ARB, *n* (%)	390 (47%)	376 (51%)	430 (58%)	412 (55%)	**<0.001**
Statins, *n* (%)	283 (34%)	307 (42%)	337 (46%)	326 (44%)	**<0.001**
Diuretic, *n* (%)	181 (22%)	191 (26%)	260 (35%)	321 (43%)	<0.001
Number of deaths					
All-cause mortality	110 (13.1%)	134 (18.2%)	146 (19.8%)	238 (31.8%)	—
Cardiovascular mortality	30 (3.6%)	39 (5.3%)	48 (6.5%)	63 (8.4%)	—

^*∗*^MI: myocardial infarction, HF: heart failure, SBP: systolic blood pressure, eGFR: estimated glomerular filtration rate, CKD: chronic kidney disease, UACR: urinary albumin to creatinine ratio, CRP: C-reactive protein, ACE: angiotensin convertase enzyme inhibitor, and ARB: angiotensin receptor blockers.

**Table 2:** Unadjusted and adjusted odds of underlying diabetes-related complications by RDW quartile.

	MI	Stroke	CKD	Retinopathy
	Odds ratio (95% confidence interval), *P* value			
Unadjusted				
Q2 versus Q1	1.72 [1.19–2.51], **P** = 0.004	1.44 [0.98–2.13], *P* = 0.063	1.69 [1.28–2.22], **P** < 0.001	1.06 [0.84–1.34], *P* = 0.64
Q3 versus Q1	2.59 [1.82–3.68], **P** < 0.001	2.16 [1.50–3.10], **P** < 0.001	2.14 [1.64–2.80], **P** < 0.001	1.03 [0.81–1.31], *P* = 0.80
Q4 versus Q1	3.82 [2.73–5.36], **P** < 0.001	2.76 [1.94–3.91], **P** < 0.001	3.76 [2.92–4.85], **P** < 0.001	1.20 [0.95–1.51], *P* = 0.14
Adjusted^||^				
Q2 versus Q1	1.55 [1.05–2.30], **P** = 0.027	1.22 [0.81–1.82], *P* = 0.35	1.20 [0.88–1.65], *P* = 0.25	0.99 [0.77–1.28], *P* = 0.96
Q3 versus Q1	2.15 [1.48–3.12], **P** < 0.001	1.73 [1.18–2.55], **P** = 0.005	1.24 [0.91–1.70], *P* = 0.17	0.90 [0.69–1.18], *P* = 0.45
Q4 versus Q1	3.17 [2.17–4.64], **P** < 0.001	1.93 [1.30–2.86], **P** = 0.001	1.64 [1.20–1.11], **P** < 0.001	0.84 [0.64–1.12], *P* = 0.23

^||^Adjusted for age, gender, race, hemoglobin, SBP, smoking, cholesterol, and insulin use. MI: myocardial infarction and CKD: chronic kidney disease.

**Table 3:** Association between RDW and all-cause and cardiovascular mortality.

	All-cause mortality		CV mortality	
	HR (95% CI)	*P* value	HR (95% CI)	*P* value
Unadjusted				
Q2 versus Q1	1.54 [1.19–1.98]	**0.001**	1.64 [1.02–2.65]	**0.041**
Q3 versus Q1	1.79 [1.40–2.29]	**<0.001**	2.16 [1.37–3.41]	**0.001**
Q4 versus Q1	3.44 [2.74–4.32]	**<0.001**	3.34 [2.16–5.17]	**<0.001**
Per 1%	1.20 [1.16–1.23]	**<0.001**	1.15 [1.08–1.23]	**<0.001**
Model 1^||^				
Q2 versus Q1	1.20 [0.93–1.55]	0.16	1.25 [0.77–2.02]	0.37
Q3 versus Q1	1.35 [1.05–1.74]	0.02	1.57 [0.99–2.50]	0.058
Q4 versus Q1	2.37 [1.85–3.03]	**<0.001**	2.22 [1.39–3.55]	**0.001**
Per 1%	1.16 [1.11–1.20]	**<0.001**	1.10 [1.01–1.19]	**0.032**
Model 2^*Ϯ*^				
Q2 versus Q1	1.17 [0.89–1.54]	0.27	1.23 [0.74–2.06]	**0.43**
Q3 versus Q1	1.25 [0.94–1.65]	0.12	1.37 [0.81–2.32]	0.24
Q4 versus Q1	2.03 [1.54–2.68]	**<0.001**	1.96 [1.16–3.31]	**0.012**
Per 1%	1.14 [1.09–1.20]	**<0.001**	1.09 [0.98–1.20]	0.13
Model 3^*ǂ*^				
Q2 versus Q1	1.26 [0.68–2.35]	0.47	1.30 [0.78–2.19]	0.32
Q3 versus Q1	1.66 [0.91–3.04]	0.098	1.41 [0.83–2.38]	0.21
Q4 versus Q1	2.39 [1.30–4.38]	**0.005**	1.99 [1.17–3.37]	**0.011**
Per 1%	1.09 [0.99–1.22]	0.094	1.08 [0.97–1.20]	0.15

^||^Model 1: age, gender, race, and hemoglobin. ^*Ϯ*^Model 2: Model 1 + HF, MI, stroke, malignancy, CKD, BMI, SBP, and cholesterol. ^*ǂ*^Model 3: Model 2 + oral antidiabetics, insulin, statins, ACE/ARBs, and diuretics.
